# Cytotoxicity and cell migration evaluation of a strontium silicate-based root canal sealer on stem cells from rat apical papilla: an in vitro study

**DOI:** 10.1186/s12903-024-04774-w

**Published:** 2024-08-30

**Authors:** Guanglei Zhou, Yu Zhao, Liangjing Cai, Liwei Liu, Xu Li, Lu Sun, Jiayin Deng

**Affiliations:** 1 School and Hospital of Stomatology, Tianjin Medical University, 12 Observatory Road, Tianjin, 300070 China; 2 Tanggu Stomatological Hospital, No. 171; Yongjiu Street, Binhai New Area, Tianjin, 300450 China; 3 Department of Periodontics and Oral Medicine, University of Michigan School of Dentistry, 1011 N University Ave, Ann Arbor, MI 48104 USA

**Keywords:** CRoot SP, Cytotoxicity, Cell migration, Stem cells from apical papilla

## Abstract

**Background:**

Calcium silicate-based bioceramics have been applied in endodontics as advantageous materials for years, many chemical components and new synthesizing methods were used to improve the base formulation of the materials for positively affecting the sealers properties. Recently, a novel biomaterial formulation, grounded in strontium silicate, has been introduced to the market, offering potential advancements in the field.

**Objective:**

To comparatively analyze the cytotoxicity and cell migration effects of a novel strontium silicate-based bioceramic material (CRoot SP) and those of calcium silicate-based (iRoot SP) and epoxide amine resin (AH Plus) sealers on stem cells derived from rat apical papilla(rSCAPs).

**Methods:**

rSCAPs were isolated and characterized in vitro and subsequently cultured in the presence of various concentrations of CRoot SP, iRoot SP and AH Plus extracts. Cytotoxicity was assessed by CCK-8 assay, and cell-migration capacity was assessed by using wound healing assays .

**Results:**

No significant differences in cell viability were observed in the 0.02 mg/mL and 0.2 mg/mL sealer groups. The cell viability of CRoot SP was consistently greater than that of iRoot SP at concentrations of 5 mg/mL and 10 mg/mL across all time points. Maximum cytotoxic effect was noted on day 5 with 10 mg/mL AH Plus.The scratch was partly healed by cell migration in all groups at 24 h, and the 0.02 mg/mL, and 0.2 mg/mL CRoot SP exerted beneficial effects on rSCAPs migration.

**Conclusions:**

CRoot SP exhibited less cytotoxic than the iRoot SP and AH Plus extracts after setting. A lower concentration of CRoot SP thus promotes the cell migration capacity of rSCAPs, and it may achieve better tissue repair during root canal treatment.

## Introduction

In recent years, calcium silicate cement sealers(CSCS) have been used as root canal sealers because of their excellent physical, chemical and biological properties [1]. The first endodontic CSCS introduced in 2007 was iRoot SP (Innovative BioCreamix, Vancouver, Canada), it is a premixed, injectable hydraulic cement paste developed for permanent root canal obturation [2]. The composition of sealers are presented in Table 1. iRoot SP identical products can be purchased under different brand names according to the sales region (Endosequence BC Sealer, Brasseler USA, Savannah, USA)(Total Fill BC Sealer, FKG Dentaire, La Chaux-de-Fonds, Switzerland). iRoot SP stimulates osteogenic differentiation of MG63 cells, human tooth germ stem cells, and human periodontal ligament stem cells (hPDLSCs). These characteristics confirm its outstanding biocompatibility and mineralization effect when used for root filling [3–5]. However, its high solubility remains a challenging issue [6]. iRoot SP sealers demonstrate high levels of calcium ion release on account of its high solubility [7], and the excessive release of calcium ions can inhibit cell proliferation and increases the cellular apoptotic rate [8, 9].

AH Plus (Dentsply International Inc, York, PA, USA) is an epoxy resin-based sealer that exhibits various beneficial properties, including dimensional stability, low solubility, adherence to the dentin, and high radiopacity. This sealer serves as the “gold standard” for the evaluation and comparison of different types of sealers. Nonetheless, AH Plus has certain limitations, such as possible mutagenicity, cytotoxicity, and hydrophobicity, which means that it should be set in a dry environment; however, the root canal is hydrophilic [10].

Sealers may extrude into the surrounding supporting tissues to varying extents during treatment [11]. The incidence of sealer extrusion is related to apical diameter, operator approach, root canal preparation, filling techniques and the sealer selected for use. Apical extrusion of premixed CSCS ranged from 11.8 to 59.8%, while apical extrusion of AH Plus or powder-liquid CSCS ranged from 11.8–33.3% [12]. The extrusion of sealers may incite a localized inflammatory response within the periradicular tissues, which may manifest as the clinical symptom of postoperative pain and/or swelling. AH plus has shown high intensity post obturation pain after unintentional extrusions. This due to the toxic effect of AH Plus.The apical extrusion of CSCS presents low-intensity postoperative pain [13]. However, one study found that the pain intensity of EndoSequence BC Sealer did not mitigate for 1 week. The properties of EndoSequence BC, characterized by longer setting time and higher solubility, contribute to the retention of its irritating constituents. Consequently, this retention perpetuates the production of inflammatory mediators, potentially prolonging the inflammatory response within the periradicular tissues [14]. During healing and repair stage, the process of resorbing extruded sealers commences. Sealers may be solubilized in the periradicular tissue fluids, phagocytosed, or encapsulated by fibrous connective tissue [15]. The ultimate destiny of the sealers depend on its physicochemical properties, especially the solubility [16]. The zinc oxide eugenol-based sealers may be complete dissolution, but only 15% of the AH Plus cases showed complete removal of the extruded material in periods longer than 4 years. The low solubility of AH Plus introduces a persistent presence, where the epoxy resin could be the culprit responsible for the sluggish rates [12]. Ca(OH)_2_-based sealers showed faster removal than resin sealers [15]. Limited information is reported regarding apical extrusion modification on CSCS. A recent investigation documented that approximately 10% of Endosequence BC extrusions vanished from radiographs after a 36-month observation period [17]. In parallel, a contemporary prospective clinical trial revealed that one-half of apically extruded Ceraseal extrusions were no longer discernible via radiographs by the 24-month mark [18]. The dissolution of extruded CSCS may be attributed to the higher solubility and longer setting time [19]. In efforts to prevent accidents and complications through the extrusion of root canal sealers, it is crucial to carefully select materials that possess desirable physicochemical properties and minimal toxicity.

Strontium (Sr) has attracted much attention owing to its positive effects on bone metabolism by preventing bone resorption and enhancing new tissue growth [20]. A new strontium silicate-based bioceramic sealer (Beijing C-root Dental Medical Devices Co. Ltd China), CRoot SP, was designed to promote fast bone regeneration and enhance healing and clinical success. It is also a a premixed, injectable hydraulic cement paste. Its biological effects must be evaluated before clinical use. Mesenchymal stem cells (MSCs) are routinely employed to repair inflammatory tissue damage because of their multiple differentiation abilities and anti-inflammatory properties [21]. Stem cells from the apical papilla (SCAPs) are a type of dental MSCs first reported by Sonoyama et al. [22]. SCAPs are considered suitable for stem cell-based regeneration, such as pulp-dentin tissue, periodontal tissue, bone and neural regeneration, owing to their high proliferative potential [23, 24]. In this study, we investigated the impact of CRoot SP on the cytotoxicity and migration of stem cells from rat apical papilla(rSCAPs) was evaluated by comparing it with those of iRoot SP and AH Plus. The benefits and limitations of CRoot SP were identified, thereby facilitating evidence-based decision-making in endodontic procedures.

**Table 1:** Composition of sealers used in this study

Sealers	Manufacturer	Components
AH-Plus	Dentsply International Inc., York, PA, USA	Paste A: bisphenol-A epoxy resin, bisphenol-F epoxy resin, calcium tungstate, ziroconium oxide, silica, iron oxide plgments Paste B: dibenzyldiamine, aminoadamantane tricyclodecane-diamine, calcium tungstate, zirconium oxide, silica silicone oil
iRoot SP	Innovative Bioceramix Inc., Vancouver, Canada	Tricalcium silicates, dicalcium silicates, calcium phosphate monobasic, calcium hydroxide, zirconium oxide, fillers, and thickening agents
CRoot SP	Beijing C-Root Dental Medical Devices Co. Ltd, China	Strontium silicate, calcium phosphate, calcium hydroxide, zirconium dioxide, fillers and thickening agents

## Materials and methods

### Isolation and culture of rSCAPS

All protocols were carried out in accordance with the guidelines of the Animal Experiment Committee of Tianjin International Joint Academy of Biomedicine. Sprague-Dawley rats (Harlan Sprague-Dawley, Beijing, China), aged 2–3 weeks were euthanized with 20% urethane (i.p., 7mL kg^− 1^) before tissue harvest. The mandibular molar teeth were carefully extracted, and rinsed with sterile phosphate-buffered saline (PBS) (Sangon Biotech, Shanghai, China). Subsequently, the apical papilla were separated from the root apex, washed with PBS, minced into small pieces, and digested with 3 mg/mL collagenase and 4 mg/mL dispase II at 37 °C for 1 h. The cell suspensions were centrifuged at 1200 rpm for 5 min and resuspended and cultured in complete DMEM (KeyGEN BioTECH, Jiangsu, China) supplemented with 15% fetal bovine serum containing 80 µg/mL streptomycin and 80 U/mL penicillin. These cells were defined as P0 and were cultured to the 3rd−5th passages for this study.

### Flow cytometry

rSCAPs were trypsinized and resuspended in PBS. The cell density was adjusted to 1 × 10^6^ cells/mL and the cells were incubated with fluorescence-conjugated monoclonal antibodies (CD24,CD34, and CD146)(Bioss Antibodies, Beijing, China) for 50 min in the dark at room temperature. Following two PBS washes, the expression profiles were analysed using a flow cytometer.

### Immunofluorescence

rSCAPs were seeded onto glass coverslips at a density of 1 × 10^6^ cells/well, fixed with 4% paraformaldehyde, and permeabilized with 0.5% Triton X-100 for 30 min at room temperature. The permeabilized cells were blocked with 1% bovine serum albumin for 1 h and subsequently incubated with primary antibodies for CD24, CD146, vimentin, and keratin (Bioss Antibodies) overnight at 4°C in the dark. This step was followed by incubation with the specified fluorescence-conjugated secondary antibodies. 4’,6-Diamidino-2-phenylindole (DAPI) mounting medium was added for nuclear counterstaining and images were acquired using an inverted fluorescence microscope.

### Preparation of the conditioned medium

CRoot SP and iRoot SP are presented in an injectable syringe format, and AH Plus is presented in a two-component paste format. Therefore, pastes A and B were mixed following the manufacturer’s instructions. The materials were solidified separately at 37 °C in a 100% humidified atmosphere with 5% CO_2_ for 3 days and dried for one day. after which they were ground into powder and filtered through a 45 μm strainer. The cells were disinfected with ultraviolet light for 1 h, immersed in 10 ml of DMEM at a 20 mg/ml concentration and incubated for 3 days at 37 °C to generate bioactive compounds. Later, the extracts were collected and filtered through 0.22 μm sterile filters. The extracts were subsequently diluted to the desired final concentrations (0.02, 0.2, 2, 5, and 10 mg/mL) with complete medium and stored at 4 °C until use.

### Cell cytotoxicity assay

Cell viability was evaluated using CCK-8 assays. A total of 4 × 10^3^ rSCAPs cells/well were seeded in 96-well plates and incubated in complete medium for 24 h. The culture medium was replaced with media supplemental with varying concentrations of CRoot SP, iRoot SP or AH plus extracts, and the cells were cocultured for another 1, 3, or 5 days. On days 1, 3, and 5, the medium was replaced with 10% CCK-8 solution (APExBIO, USA) for 2 h at 37 °C under 5% CO_2_. The optical density values were read at 450 nm.

### Wound healing assay

A wound healing assay was performed to assess the migration of rSCAPs in vitro. Cells were seeded in six-well plates and 200 µl sterile pipette tips were used to create a central scratch through the monolayer of cells. Cell debris was removed by washing the cells twice with PBS. The cells were maintained in DMEM supplemented with 1% FBS and 0.02, 0.2, or 2 mg/mL CRoot SP, iRoot SP, or AH Plus extract respectively. The wound areas were measured at 0 and 24 h using a phase contrast microscope equipped with a digital camera, and the wound widths were quantified using ImageJ software.

### Statistical analysis

Statistical analysis was performed using SPSS Statistics 21 software. All experiments were repeated thrice for data analysis, and the results are expressed as the mean ± standard deviation. Cell viability and the percentage of wound healing were determined using a one-way analysis of variance, followed by least significant difference testing. The results were considered significant if *p* < 0.05.

## Results

### Characterization of rSCAPs

**Fig. 1:**
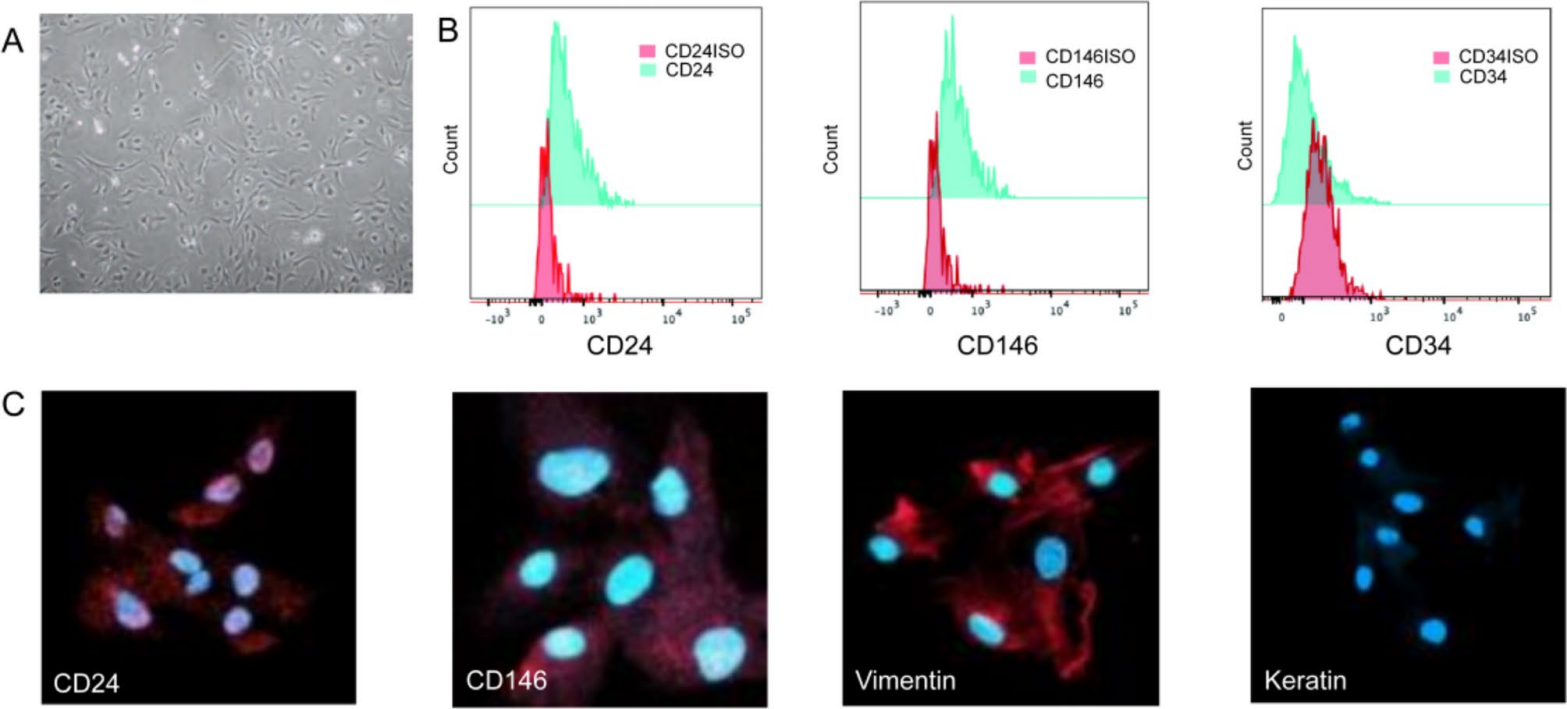
Isolation and characterization of stem cells from rat apical papilla (rSCAPs). **A** Primary culture of rSCAPs. **B** Flow cytometry analysis of rSCAP surface markers. The histogram represents the cell count on the Y-axis and the fluorescence intensity on the X-axis. Histogram overlays of unstained control cells (red histogram) and cells stained with antibodies against the surface proteins CD24, CD146 and CD34 (green histogram). **C** Characterization of rSCAPs by immunofluorescence staining for CD24, CD146, vimentin and keratin

As depicted in Fig. 1, the isolated rSCAPs were spindle-shaped and exhibited a clear outline, with stable growth and passage. Flow cytometry analysis revealed that the cells expressed MSC surface markers, including CD24 and CD146, but lacked CD34, a haematopoietic stem cell surface marker. CD24 is a specific marker for SCAPs and is not detectable in other MSCs. Immunofluorescence staining of the SCAPs signified that the cells positively expressed CD24, CD146, and vimentin but not keratin. Based on these observations,,the isolated cells were confirmed to be SCAPs .

### Cell viability

**Fig. 2:**
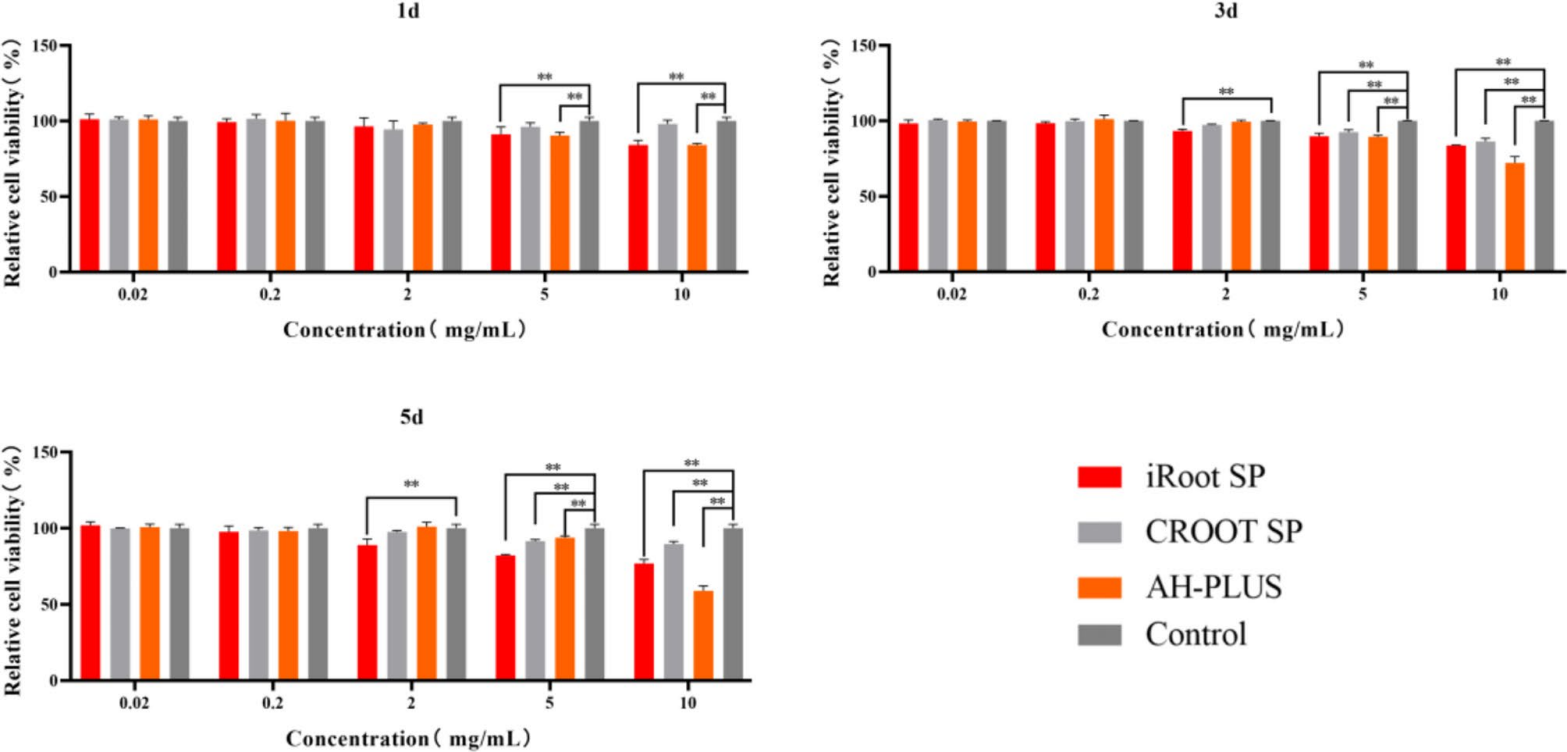
The viability of rSCAPs treated with different sealer extracts for the indicated times, as determined by a CCK-8 assay. P values were significantly different between the selected groups (*p < 0.05; **p < 0.01)

CCK-8 assays were performed to determine the viability of the rSCAPs. As illustrated in Fig. 2, compared with the control group, significant differences in cell viability were not detected at concentrations of 0.02 and 0.2 mg/mL for the three types of sealers and at 2 mg/mL for CRoot SP and AH Plus (*p* > 0.05). Conversely, cells treated with iRoot SP and AH-Plus at concentrations of 5 and 10 mg/mL exhibited significantly lower viability than the control group(*P* < 0.05). On day 1, the cell viabilities of plants treated with CRoot SP (5 and 10 mg/mL) and iRoot SP (2 mg/mL) was comparable to those of the control; however, they were significantly reduced on days 3 and 5 (*P* < 0.05). Moreover, the cell viabilities of CRoot SP was consistently greater than that of iRoot SP at 5 and 10 mg/mL across all time points. The maximum cytotoxic effect was observed on day 5 with 10 mg/mL AH Plus.

### Cell migration

**Fig. 3:**
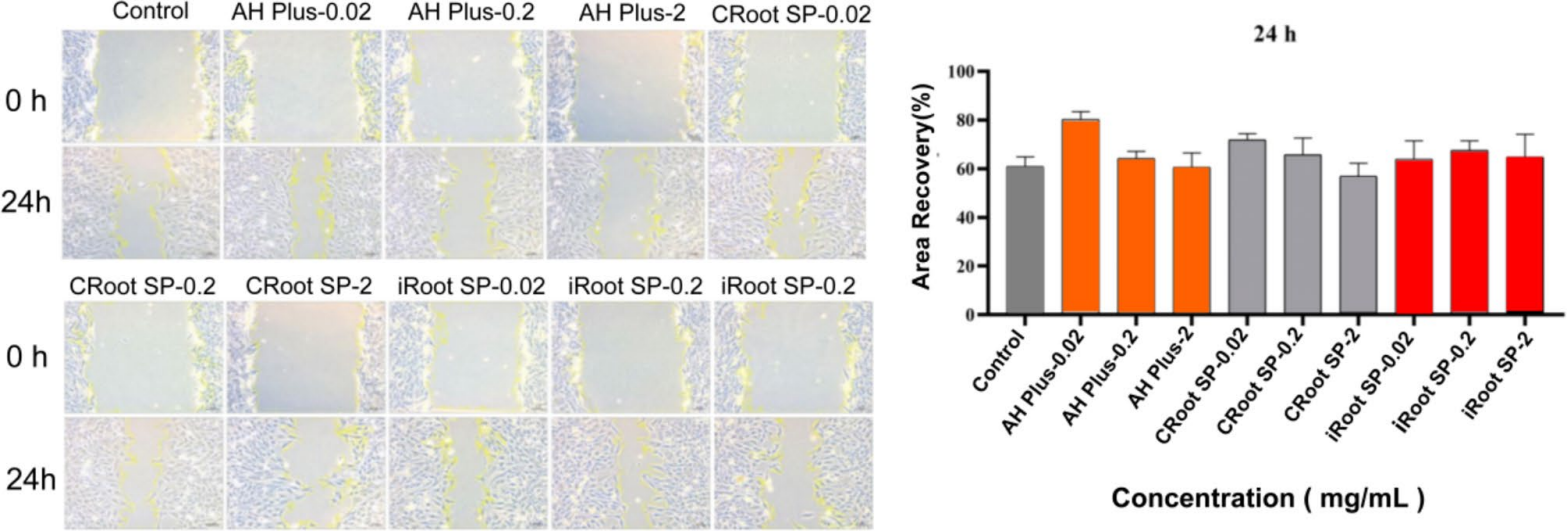
Wound healing assays showing the effect of different concentrations of different sealer extracts on the migration of rSCAPs for up to 24 h. The area recovery percentage of rSCAPs by wound healing assays on three types of sealer extracts at 24 h

The impact of the sealers on the migration of the rSCAPs was evaluated using the wound healing assay. As presented in Fig. 3, the scratches were partially healed by cell migration in all the material groups as well as the control at 24 h, and the differences were not statistically significant (*p* > 0.05). In the AH Plus and CRoot SP groups, compared with 0.2 and 2 mg/mL, 0.02 mg/mL exhibited superior cell migration, and the migration ability decreased as the concentration increased. In the iRoot SP group, the cell migration ability of 0.2 mg/mL was better than that of 0.02 and 2 mg/mL. CRoot SP positively influences rSCAPs migration at appropriate concentrations.

## Discussion

The periapical disease is characterized by local inflammation, bone and tissues destruction, root canal therapy is one of the most effective treatments for periapical diseases, many studies are being focused on developing novel clinical strategies to repair the apical bone defects. Sealers might impact periapical tissues either via direct contact or the release of components. These effects can alter the microenvironment and influence stem cell viability, migration and differentiation in apical tissue [25]. The ideal sealer should be sufficiently biocompatible and not cytotoxic. Cytotoxicity assessments using various cell lines, laboratory methods, concentrations and dilutions, and sealer setting conditions (freshly mixed or set) contribute to the complexity of interpreting results [26]. It is well-documented that all sealers exhibit some level of toxicity in their fresh state, which significantly diminishes after setting [27]. For instance, AH Plus has been reported to be severely cytotoxic when it is fresh [28], but becomes relatively inert once cured [29]. From a clinical perspective, the use of freshly mixed sealers is relevant to accsee the cytotoxicty due to their application in an unset condition within root canals. The extruded sealers couldn’t be complete dissolution in fact. Therefore, the cytotoxicity of sealers should also be assessed in the set condition, and obtaining material extracts after curing can help control variations to some extent for each testing material [30]. At present, most bioceramic sealers are based on calcium silicate (CaSi). Two forms of CaSi sealers are commercially available: manually mixing sealers (powder-liquid) and ready-to-use sealers (premixed) [12]. The powder/liquid sealers demand precise manual mixed following the manufacture’s instructions, leaving no room for deviation in the powder-to-liquid ratio. Such deviation can lead to compromises in the sealer’s compressive strength, solubility, and its adhesive bond with dentin [31]. To improve manipulability and ease of application, the premixed CSCSs have been developed. These sealers prove to be easier to perform and have a short learning time [32]. CaSi is the bioactive composition of the material. Presently, a multitude of premixed CSCSs populate the market, with their primary compositions detailed within Table 2. They have different percentages of CaSi and different radiopacifiers in their composition.The varying percentages of CaSi confer different chemical, physical and biological properties [12].The sealers with higher amount of CaSi exhibited enhanced cumulative calcium release. This attribute not only bolsters the acid resistance and structural integrity of dentin but also precipitates apatite early on which can enhance the biointeractive and biological properties when applied in periapical bone defects [33]. However, they also show higher solubility which lead to poor sealing ability and affect the long-term antibacterial performance. This issue is particularly prevalent in sealers dominated by tricalcium silicate [34]. A low amount of CaSi in the sealer formulation also means lower silanol (Si-OH) functional groups, which are instrumental in promoting apatite nucleation [33]. The percentage and types of radiopacifiers may influence their appearance in the root canal and periapical area radiographically [12]. Heavier atoms confer greater radiopacity, with the following agents tending to exhibit diminishing levels of this property: bismuth oxide (Bi_2_O_3_), zirconium oxide (ZrO_2_), calcium tungstate, barium sulfate (BaSO_4_), and zinc oxide [2]. Bi_2_O_3_ and ZrO_2_ are commonly used as radiopacity agents for root canal sealers. However, Bi_2_O_3_ will influence the hydration, mechanical properties of the sealer and can cause discoloration of teeth. ZrO_2_ is more biocompatible than Bi_2_O_3_ and BaSO_4_. The integration of ZrO_2_ into the premixed sealer had no adverse effect on the flowability, film thickness, solubility, injectability and washout resistance [35]. The premixed CSCS have faced criticism due to their extended setting time and higher in vitro solubility [12]. New bioceramics were developed to overcome the disadvantages of the premix CSCS such as Calcium aluminate cement (Dia-Root Bio Sealer, DiaDent, Cheongju, Republic of Korea), which exhibited higher push-out bond strength and degree of biomineralization than Endoseal MTA and EndoSequence BC [36]. CRoot SP is a recently introduced biomaterial formulation based on strontium silicate. Zhang found strontium silicate could induce hydroxy carbonate apatite formation in simulated body fluid [37], which is more close to the hydroxyapatite in natural bones in term of composition. As the intracellular transport pathway of Sr is quite similar to that of calcium (Ca), Sr ions exhibit a strong affinity for binding to the bone matrix during mineralization. Sr alleviates osteoclast activity and bone resorption in vitro, an effect that is not observed with Ca [38]. Furthermore, Sr exerts immunomodulatory effects, which provides an appropriate environment for augmenting bone regeneration [39]and its weight is higher than Ca, which enhances the radiopacity [40]. The premixed, injectable sealer eliminates the potential for heterogeneous consistency during on-site mixing. In addition, it is convenient to get the apex during root canal filling for treating bone defects by concentrating the Sr ions at the defect site to facilitate the bone healing process. Yan et al. reported that strontium silicate-based sealers promote the proliferation and osteogenic differentiation of hPDLSCs in vitro [41]. Yang et al. showed that CRoot SP exhibits low cytotoxicity, certain osteogenic potential on L929 and MC3T3-E1 cells, and possesses antibacterial activity against Enterococcus faecalis [42]. This study aims to compare the cytotoxicity and cell migration ability of CRoot SP, iRoot SP, and AH Plus on rSCAPs.

**Table 2:** Composition of the premixed calcium silicate sealers

Sealer	Manufacture	composition
EndoSequence BC Sealer	Brasseler, Savannah, GA, USA	Tricalcium silicate (20–35%), dicalcium silicate (7–15%), calcium hydroxide (1–4%),zirconium oxide (35–45%) as radiopacifier
Bright Endo MTA sealer	Genoss, Suwon, Korea	Calcium silicates(50–70%),methyl cellulose(20–30%) ,bismuth oxide(10% ) as radiopacifier
Ceraseal	Meta Biomed Co. South Korea	Tricalcium silicate (20–30%),dicalcium silicate (1–10%), tricalcium aluminate (1–10%), zirconium dioxide (45–50%) as radiopacifiers, thickening agents
Endoseal MTA	Maruchi, Wonju, Korea	Natural pure cement[calcium silicates, calcium aluminates, calcium aluminoferrite, and calcium sulfates] (27.81%), zirconium dioxide/bismuth trioxide (47.28%)as radiopacifier, thickening agents (24.91%)
NeoSealer Flo	Avalon Nusmile, USA	Tricalcium silicate (< 25%), dicalcium silicate(< 10%), calcium aluminate (< 25%),calcium aluminum oxide (< 6%), tricalcium aluminate (< 5%),tantalite (50%) as radiopacifier
AHPlus Bioceramic	Dentsply, Konstanz, Germany	Tricalcium silicate (10–15%),zirconium dioxide (50–70%) as radiopacifier, thickening agents
Nano-Ceramic Sealer	B&L Biotech, Fairfax, USA	Calcium silicates, zirconium oxide, filler, thickening agent
Well-Root ST	Vericom, Gangwon-Do, Korea	Calcium silicate compound, calcium sulfate dehydrate, calcium sodium phosphosilicate, zirconium oxide, titanium oxide, thickening agents
Bio-C Sealer Ion+	Angelus, Londrina, PR, Brazil	Calcium silicate, magnesium silicate, calcium sulphate, potassium sulphate, zirconium oxide, silicon dioxide and dispersing agent
BioRoot™ Flow	Septodont, Saint Maur Des Fosses, France	Tricalcium silicate, propylene glycol, povidone, calcium carbonate, aerosil (silica), zirconium oxide, acrylamide/sodium acryloyldimethyltaurate copolymer, isohexadecane and polysorbate
Sealer Plus BC	MK Life, Porto Alegre, Brazil	Tricalcium silicate, dicalcium silicate, calcium hydroxide and propylene glycol, zirconium oxide
BrightEndo MTA sealer	GENOSS, Suwon, Korea	Calcium silicates, zirconium oxide, bismuth oxide, solvent/thickening agent
Endoseal TCS	Maruchi, Wonju, Korea	Tricalcium silicate, phyllosilicate mineral, zirconium oxide, dimethyl sulfoxide
Sureseal	Sure Dent, Gyeonggi-do, Korea	Calcium silicates, calcium phosphate, calcium hydroxide, filler, and thickening agents
Total Fill BC Sealer HiFlow	FKG Dentaire, Switzerland	Dicalcium silicate, tricalcium silicate, calcium hydroxide, fillers, zirconium oxide

The MTT assay has become the standard test for assessing cytotoxicity, however, compared with MTT, the CCK-8 assay provides superior detection sensitivity and accuracy [43]. In this study, CCK-8 assay results signified that cell viabilities were comparable at lower concentrations of all sealer extracts and the control, and the maximum cytotoxic effect was observed on day 5 for 10 mg/mL AH Plus. AH Plus was introduced to overcome the shortcomings of its precursor AH26, especially formaldehyde release [44]. The manufacturer claims that AH Plus is a formaldehyde-free material; but a study has reported the release of 3.9 ppm of formaldehyde from it [45]. The material’s severe toxicity could be attributed to its amine content [44] and epoxy resin component [46]. These potentially toxic constituents are not completely or partially converted post setting, as a result of which AH Plus exhibits residual toxicity [47] in a concentration- and time-dependent manner [48], with higher concentrations showing increased cytotoxicity after setting.

Sealer solubility can trigger the release of chemical compounds that can possibly irritate periapical tissues. One major limitations of CSCSs is their high solubility [49], which is associated with the formation of soluble calcium salts and calcium hydroxide during the reactions [50]. Calcium hydroxide dissociates into calcium and hydroxyl ions(OH ^−^). Part of the dissolution of Ca ions lead to the formation of a negatively charged surface with the silanol functional group. Silanol groups (Si-OH) bind calcium or phosphate ions through electrostatic interaction, triggering the nucleation of apatite [51]. Calcium can impact various aspects of cell function, including biological behavior and apoptosis [52]. Maeno et al. [53] found that low (2–4 mM) and medium (6–8 mM) concentrations of calcium are appropriate for osteoblast proliferation, differentiation, and mineralization, high concentrations (> 10 mM) concentrations (> 10 mM) are cytotoxic. High amounts of the ion can disrupt calcium homeostasis around the cells, and negatively affect cellular metabolism, potentially leading to cell apoptosis [54]. The solubility of iRoot SP is high (20.64%), increasing over time and exceeding the American National Standards Institute/American Dental Association tolerance of 3% by mass [7]. This high solubility is due to hydrophilic nanosized particles, which increase their surface area and allows more liquid molecules to come into contact with the sealer [55]. iRoot SP has been reported to have a significant leaching of calcium, particularly pronounced within the first 14 days [7]. Another major limitation of CSCSs their high alkalinity, with a pH ranging between 10 and 12 for weeks after setting. iRoot SP exhibited a high alkaline pH both before and after setting [56], with a pH value of 11.5 even after setting [57, 58]. Calcium hydroxide helps maintain alkaline conditions and contributes to hard tissue formation, neutralizes lactic acid, and eradicates microorganisms. However, excessive hydroxyl ions can denature adjacent cells and medium proteins [59]. Calcium phosphate in the cement formulation can react with Ca(OH)_2_, consuming it and forming a Calcium Silicate Phosphate Hydroxide gel. This reaction potentially reduces the content of calcium hydroxide and the alkalinity of the cement [60]. Studies have shown that iRoot SP was more cytotoxic than Pro-Root MTA in RAW 264.7 cells in a dose-dependent manner [61]. Zhang et al. reported that iRoot SP was slightly cytotoxic to L929 cells after 24 h, which they attributed to the high surface pH and the release of a large amount of hydroxyl ions [59]. Loushine et al. found that iRoot SP exhibited severe cytotoxicity on MC3T3-E1 mouse osteoblasts after 24 h and remained moderately cytotoxic over a 6-week period [62]. In this study, the cytotoxicity of iRoot SP increased with the dose and duration, with 2 mg/mL iRoot SP being more cytotoxic than 2 mg/mL CRoot SP and AH Plus by day 3, This could be due to the high alkalinity and the elevated concentrations of calcium, creating a hostile environment in the medium as the extract concentration increased.

Strontium and calcium belong to the same main group in the periodic table and have similar chemical structures and properties. However, biological differences between the two elements exist, partially due to the larger size of the Sr molecule. These difference are evident in processes involving active transport across biological membranes, where Ca is transported more readily than Sr [63]. According to the manufacturer, the solubility of CRoot-SP was 0.49, and the pH was 12 in vitro. This suggests that a higher concentration of CRoot SP may result in the release of fewer ions. These factors could explain why 5 and 10 mg/mL of CRoot SP did not exhibit cytotoxicity on day 1 and why the cell viability percentages of cells exposed to CRoot SP were greater than those exposed to iRoot SP at 5 and 10 mg/mL across all application times in the study.

The wound healing assay evaluates both cell proliferation and migration capacities. The average population doubling time for MSCs is approximately 2 days [45]. Consequently, in this study, the observation time point for wound healing closure was set at 24 h, which is considered the time at which rSCAPs migrated. The findings revealed that 0.02 mg/mL AH Plus displayed the greatest effect on cell migration, which decreased as the concentration of AH Plus increased in the AH Plus group. These observations are in line with Rodríguez-Lozano’s study, in which immersion with different dilutions of AH Plus extracts for 24 h promoted cell migration to a level that was similar to that of the control [64]. AH Plus exhibits limited cytotoxicity after setting, and lower concentrations of the material contain fewer cytotoxic components, such as resin [65], which can negatively influence cell migration. As the concentration of AH Plus increased, the amount of resin also increased, thereby leading to a decrease in the cell migration ability.

In the CRoot SP group, the cell migration ability of 0.02 mg/mL CRoot SP was the best, and decreased with increasing concentration. Sr is a trace element in the human body, and the extracellular fluids contain only micromolar concentrations of it. Even minor alterations in strontium levels can significantly affect cellular behavior. Sr concentrations in the range of 3.75 × 10^− 3^– 0.12 mM were found to exert beneficial effects on the cellular response, whereas those > 0.12 mM exhibited detrimenta impacts on cells [37]. This finding indicates that an optimal Sr concentration range exists. Although the precise concentration of Sr in the medium of the CRoot SP group was not measured in this study, it might have exceeded the optimal level as the concentration increases, potentially leading to a negative effect on cell migration.

In the iRoot SP group, cell migration at a concentration of 0.2 mg/mL was greater than that at 0.02 and 2 mg/mL.Wu et al. reported that the migration ability of stem cells from human apical papilla cultured with 0.2 mg/mL iRoot SP extract was greater than that of those cultured with 0.02 and 2 mg/mL iRoot SP extract [66]. The migration ability of hPDLSCs stimulated with 5 mmol/L and 10 mmol/L Ca^2+^ was significantly enhanced compared with that of the control group, whereas treatment with 15 mmol/L Ca^2+^ reduced the migration ability [67]. This observation alludes that Ca^2+^ promotes cell migration within an appropriate range and the cell migration ability increases with increasing Ca^2+^ concentration. However, beyond this range, Ca^2+^ has no positive effect on cell migration and may, in fact, impede it. As the concentration of iRoot SP increases, resulting in increased Ca^2+^ release, the effect on migration ability varies accordingly.

Our study has several limitations. First, the biological behaviors of experimental cells isolated from mice may be different from those of human cells. Second, endodontic sealers are clinically applied in a fresh state, which accurately reflects the clinical environment, However, in this study, materials were tested in a set state. Third, CRoot SP is a newly introduced premixed material, and only limited information is available on its filling ability, physical properties, and antibacterial activity. Finally, new sealers should be tested not only for their cytotoxicity in vitro but also for their biocompatibility in vivo before being released into the market.

## Conclusions

Within the confines of this study, it can be inferred that the novel strontium silicate-based bioceramic material, CRoot SP, demonstrates lower cytotoxicity than calcium silicate-based (iRoot SP) and epoxy resin-based sealer (AH Plus) extracts under setting conditions. Moreover, CRoot SP enhanced the migration of rSCAPs at low concentrations. Nonetheless, further in vitro and in vivo studies are required to confirm the suitability and biology of this sealer for broader clinical use.

## Data Availability

The datasets used and analysed during the current study available from the corresponding author on reasonable request.

## Abbreviations

rSCAPs: Stem cells from rat apical papilla
CSCS: Calcium silicate cement sealer
Sr: Strontium
Ca: Calcium
hPDLSCs: Human periodontal ligament stem cells
MSCs: Mesenchymal stem cells
MTA: Mineral trioxide aggregate
OH: Hydroxyl
